# Levels and predictors of knowledge of epilepsy among patients attending the epilepsy clinic of a neuro-psychiatric facility in North-Eastern Nigeria

**DOI:** 10.11604/pamj.2023.45.59.33293

**Published:** 2023-05-25

**Authors:** Mohammed Mahmood Yusuf, Ibrahim Abdu Wakawa, Falmata Baba Shettima, Asma'u Mohammed Chubado Dahiru, Umar Baba Musami, Isa Bukar Rabebbe

**Affiliations:** 1 Department of Mental Health, Federal Neuro-Psychiatric Hospital Maiduguri, Borno State, Nigeria,; 2 Department of Mental Health, College of Medical Sciences, University of Maiduguri, Borno State, Nigeria

**Keywords:** Epilepsy, predictors, knowledge, Nigeria

## Abstract

**Introduction:**

significant numbers of patients with epilepsy have poor knowledge of their disease. Patients' knowledge of disease is related to their success in coping with the disease and adherence to medication; which is one of the key factors that determined the success of pharmacotherapy in patient with epilepsy. In this study, we evaluate the level of knowledge of epilepsy among patient with Epilepsy in a tertiary mental health care facility.

**Methods:**

using a cross-sectional design, 410 patients with epilepsy attending general outpatient clinic of Federal Neuropsychiatric Hospital, Maiduguri in Northeastern Nigeria were sampled randomly into the study after fulfilling the inclusion and exclusion criteria. Socio-demographic, clinical and epilepsy knowledge questionnaires were used for data collection. Data were analyzed using the Statistical Package for Social Sciences (SPSS) version 18

**Results:**

263 (64.1%) had poor knowledge of epilepsy and 290 (70%) had less frequent seizures (having fewer than four seizure episodes in the last three months). Independent predictors of knowledge of Epilepsy were intermediate skilled employment (O.R = 2.32, P = 0.022, 95% C I = 1.13 - 4.76); semi-skilled employment (O. R = 1/85, p = 0.001, 95% C I = 1.10 - 3.12); seizure frequency (O R = 1.72, p = 0.031, 95% C I = 1.31 - 3.24).

**Conclusion:**

the level of knowledge of epilepsy among people with epilepsy was low with more than 64.1% percent of the participants had poor knowledge of epilepsy. Occupational status and seizure frequencies were independent predictors of knowledge of epilepsy. We therefore recommend psycho-educational programs be incorporated in the routine clinical care of the patients and for clinicians to pay more attention to providing information and education needs of patients.

## Introduction

Epilepsy is one of the common neurological disorders; it affects nearly 70 million people in the world [1,2]. It is estimated that 80% of People with epilepsy (PWE) live in developing countries where the treatment gap remained huge [2]. Factors such as poor obstetric services, malnutrition, childhood cerebral infections, and head trauma are possibly responsible for the high incidence [2]. Chronic conditions generally contribute hugely to Disability Adjusted Life Years (DALYs). Neuropsychiatric conditions account for approximately 28% of the DALYs due to non-communicable diseases. Epilepsy is a chronic neuropsychiatric disorder that accounts for 1% of the DALYs due to non-communicable diseases and contributes 3.57% of the burden due to neuropsychiatric conditions [3,4]. Epilepsy is a widely misunderstood disorder as it is often associated with knowledge deficits, shrouded in myth and fear as well as stigmatization. These knowledge deficits are prevalent not only in the general population but even among PWE especially in developing countries such as Nigeria [5,6]. A significant percentage of PWE do not know much about the medical aspects of their disease particularly when it comes to the aetiology, medication and outcome of the investigation, etc. This is regardless of their age, educational background, or number of years with epilepsy [7]. Furthermore, 35% of PWE report not having adequate information about their disease conditions [8]. Misinformation and erroneous beliefs among these patients and their caregivers can lead to severe medical and psychosocial consequences, e.g. misuse of Antiepileptic Drugs (AEDs), dangerous first aid practices, and unnecessary restrictions on daily life activities, poor Quality of Life (QOL), depression and social withdrawal. Poor Knowledge of Epilepsy (KOE) is also significantly associated with low self-esteem, social anxiety and stigma [9]. Similarly, poor KOE is associated with a wider treatment gap as the World Health Organization (WHO) estimates that, about 80% of PWE in Africa do not receive treatment despite effective treatment options being available [10].

Factors responsible for this low treatment rate include poor patient-provider communication, a traditional belief system about the causes of Epilepsy and lack of “medical knowledge” about the causes, symptoms, diagnoses and treatments of Epilepsy and limited financial resources [10,11]. Different African settings have varying patterns of belief systems about Epilepsy; many people from the diverse sub-Saharan African cultures believe that epilepsy is due to either the effect of witchcraft, possession by evil spirits or angered ancestral spirits [12]. Some believe that the act of foaming or drooling saliva during a seizure is contagious and this results in families being shunned and excommunicated from society [13,14]. Traditional treatments (herbal preparations, spiritual exorcism, special cultural diets, charms, and sacrificial offerings) have been seen, in most parts of sub-Saharan Africa including Nigeria, to be the most preferred treatment options for PWE [11,14]. The extent of understanding of epilepsy among its sufferers affects the psychosocial adjustment to the disease, adherence to treatment and predicts QOL of patients [15,16]. Knowing the levels of patients´ understanding of different aspects of epilepsy and its management will inform clinicians on how to structure psychoeducational interventions for their patients.

Best on the available literature searched, there is a dearth of research that looked at the predictors of KOE among its sufferers, particularly in Northeastern Nigeria; this study will therefore be the first to evaluate the socio-demographic and clinical predictors of levels of KOE among its sufferers in Northeastern Nigeria. Hence, the aim of the study is to assess the level of KOE and its socio-demographic and clinical predictors among PWE in Federal Neuropsychiatric Hospital Maiduguri, a tertiary health care facility in Northeastern Nigeria. Thus, the study will seek to answer the following research questions: 1) what is the level of knowledge of epilepsy among its sufferers attending the epilepsy clinic in Federal Neuropsychiatric Hospital Maiduguri; 2) what are the socio-demographic and clinical predictors of levels of knowledge of epilepsy among patients attending the epilepsy clinic in Federal Neuropsychiatric Hospital Maiduguri?

## Methods

**Study design, study setting, sampling technique, participants:** it was a cross-sectional study, carried out in the Epilepsy Clinic of Federal Neuro-Psychiatric Hospital Maiduguri, a specialist mental health hospital in Northern Nigeria. The clinic runs four times a week between 8 am and 4 pm with an average of 200 PWE seen in the clinic every week. The study was carried out among 410 participants; patients who fulfilled inclusion criteria were randomly sampled by the use of a table of random numbers from a total of 1,601 PWE who attended the clinic during the study period. The inclusion criteria included: patients diagnosed with Epilepsy by a consultant psychiatrist within the age range of 19 - 65 years, PWE who have been on follow-up visits and Antiepileptic Drugs (AEDs) treatments for at least 3 months and patients who have given informed consent. While exclusion criteria were: patients presenting for the first time; patients with co-morbid psychiatric conditions such as schizophrenia, intellectual disability, etc. and patients with established clinically diagnosed neurological conditions such as multiple sclerosis, established non-epilepsy-related cognitive impairment such as dementia, etc.

### Instruments

**Socio-demographic questionnaire:** a pre-designed socio-demographic questionnaire was used to collect data on the socio-demographic characteristics of the participants (age, educational status, marital status, and tribe and employment status).

**Clinical/seizure variables questionnaire:** the clinical/seizure variables questionnaire was used to assess the following: seizure type (partial, partial with secondary generalization, generalized), seizure frequency/month, seizure control for over three months (yes/no), Epilepsy duration/years and AEDs treatment (types and number of AEDs being used for treatment). Seizure frequency was assessed according to the following four categories: 0, no seizures in the last 3 months; 1, one to three seizures in the last 3 months; 2, four to six seizures in the last 3 months and 3, more than six seizures in the last 3 months [17,18]. Patients´ clinical records were used to obtain vital information such as the types of epilepsy, numbers and types of AEDs the patients were taking, EEG reports and findings, Duration of Epilepsy (DOE), Duration of Untreated Epilepsy (DOUE) and Duration of Treatment (DOT).

**Epilepsy knowledge questionnaire (EKQ):** the EKQ is a 10-item questionnaire developed by adapting previously used questionnaires by Kabir *et al*. [19] in their study in Northern Nigeria titled: “Knowledge, Attitude and Beliefs about Epilepsy among Adults in a Northern Nigerian Urban Community” [19] and that used by Ibinda *et al*. [18] in their study titled: “Evaluation of Kilifi Epilepsy Education Programme: A Randomized Controlled Trial” [18]. The questionnaire was used to assess the Knowledge of Epilepsy (KOE) among the participants. The questions were categorized into three sections: (1) causes of epilepsy, (2) prognosis of epilepsy and (3) biomedical treatment. The sections consist of 7, 2 and 1 questions respectively. The responses were recorded as ´yes´, ´no´ or ´don´t know´. Questions number 1, 6 and 10 are negatively worded questions, therefore reverse scoring system was used (i.e. a score of one was awarded for responses marked ´no´ and zero for responses marked yes or I don´t know). For the other questions, a score of one was awarded to responses marked ´yes´ and a score of zero to responses marked ´no´ or I don´t know. Participants can get a maximum score of ten (10). The score was dichotomized into ´good knowledge´ and ´poor knowledge´. Any score above the mean score of the pretest was considered good knowledge while a score below the mean was considered poor knowledge. It takes approximately 3 - 5 minutes to administer.

**Data analysis:** data were analyzed using the SPSS version 18. While descriptive statistics: mean, range and standard deviation were used to describe continuous variables, frequencies and percentages were used to analyze categorical variables. The Chi-square test was used to analyze the socio-demographic variables, clinical variables and outcome variables. Binary logistic regression was used to assess independent predictors and the knowledge of epilepsy.

**Ethical consideration:** ethical clearance was obtained from the Ethics Review Committee of Federal Neuropsychiatric Hospital Maiduguri. An informed consent form soliciting the enrollees´ participation and explaining the study protocol was given to all potential participants after assuring them of utmost confidentiality as use of only codes will be employed for the purpose of analysis and no names will be indicated on the study instruments.

## Results

**Socio-demographic characteristics of the study participants:** the study population was made up of a total of 230 males (56.1%) and 180 females (43.9%), their ages ranged from 19 to 64 years, with a mean age of 31.6 years (± 9.5). The main ethnic groups of the participants were Kanuri 193 (47.1%) accounting for almost half of the study population; Hausa 65 (15.9%), Babur 33 (8.0%), Fulani 31 (7.6%) and other tribes 88 (21.5%). Only 113 (27.6%) of the study participants had high levels of educational attainment (i.e. having ≥ 12 years of formal education); 168 (41.0 %) had only Qur´anic education, while the remaining participants had either no education, 34(8.3 %) or low levels of education (≤ 11 years of education), 95 (23.2 %). Furthermore, approximately half of the participants were unemployed and were single accounting for 187 (45.6 %) and 188 (45.9 %) respectively. Other findings on the socio-demographic characteristics are depicted in Table 1.

**Table 1:** socio-demographic characteristics of the entire study participants at baseline

Variables	Frequency (%)
N=410	
**Age group (mean ± SD) (31.58 ± 9.47 ) (19 - 61 = 42)**	
19-28	186 (45.4)
29-38	124 (30.2)
39-48	76 (18.5)
49-58	21 (5.1)
≥59	3 (0.7)
**Sex**	
Male	230 (56.1)
Female	180 (43.9)
**Educational status^a^**	
No education	34 (8.3)
Low education	95 (23.2)
High education	113 (27.6)
Qur'anic education	168 (41.0)
**Marital status**	
Single	188 (45.9)
Married	183 (44.6)
Divorced	23 (5.6)
Widowed	16 (3.9)
Othersy	0 (0.0)
**Occupational class^#^**	
Professional/skilled (class I)	1 (0.2)
Intermediate Skilled (class II)	52 (12.7)
Semi-skilled (class III)	115 (28.0)
Unskilled (class IV)	55 (13.4)
Unemployed (class V)	187(45.6)
**Ethnicity^b^**	
Kanuri	193 (47.1)
Hausa	65 (15.9)
Fulani	31 (7.6)
Babur	33 (8.0)
Others	88 (21.5)

SD: standard deviation, a = high education is having ≥ 12 years of western education, low education < 12 years of western education, # = (Boroffka & Olatawura), b = Other major ethnicity assessed included, Yoruba, Igbo, Marghi, Bolewa and Shuwa, y = for separated, widower, etc.

**Clinical characteristics of the study participants:** majority of the participants had focal seizures 223 (54.4 %) as against 187 (45.6%) who had generalized seizures. About one-half of the participants 207 (50.8%) had no seizures in the last three months prior to the study. Participants on monotherapy formed the overwhelming majority of participants 383 (93.4%) as compared to those on polytherapy 27 (6.6%). The mean age of onset of Epilepsy was (mean ± SD) (19.12 ± 1.15) (0 - 59 = 59), the mean duration of Epilepsy (mean ± SD) (12.32 ± 9.12) (0.5 - 42 = 41.50), the mean duration of the untreated seizure (mean ±SD) (5.23 ± 5.8) (0 - 32 = 32), the mean duration of treatment (mean ± SD) (7.05 ± 6.23) (0 - 40 = 40) and the mean seizure frequency (mean ± SD) (2.46 ± 3.21) (0 - 14=14) in the last 3 months. Other findings on the clinical characteristics of the participants are displayed in Table 2.

**Table 2:** clinical characteristics of all the entire study participants at baseline

Variables	Frequency (%)
N = 410	
**Class of seizure**	
Focal	223 (54.4)
Generalized	187 (45.6)
**Age of onset of Epilepsy^a^ (mean ±SD) (19.12±1.15) (0-59=59)**	
Short duration (0-15) year	174(42.4)
Medium duration (16-30)	170(41.5)
Long duration >30 years	66(16.1)
**Duration of Epilepsy (mean ± SD) (12.32 ±9.12) (0.5-42 = 41.50)**	
Short duration (0-15) year	287(70.0)
Medium duration(16-30)	107(26.1)
Long duration >30 years	16(3.9)
**Duration of untreated seizure (mean ±SD) (5.23 ± 5.8) (0 - 32 = 32)**	
Short duration (0-15) year	381(92.9)
Medium duration (16-30)	28(6.8)
Long duration >30 years	1(0.2)
**Duration of treatment (mean ± SD )(7.05 ± 6.23) (0 - 40 = 40)**	
Short duration (0-15) year	375(91.5)
Medium duration (16-30)	32(7.8)
Long duration >30 years	3(0.7)
Antiepileptic drugs combination^b^	
Monotherapy	383 (93.4)
Polytherapy	27 ( 6.6)
**Seizure frequency# (Killifi) (Mean ± SD) (2.46±3.21) (0-14=14)**	
No seizure in last 3 months	207 (50.8)
1-3 seizures in last 3 months	82 (20.0)
4-6 seizures in last 3 months	68 (16.6)
>6 seizures in last 3 months	53 (12.9)

a; 0-15 = short duration of epilepsy, 16 - 30 = medium duration, > 31 is long duration, b = majority of the patients were on Carbamazepine, # = seizure frequency categories adopted from Ibinda *et al*. (Killifi Epilepsy study, Kenya)

**Relationship between socio-demographic variables and levels KOE:** except for the occupational status (p < 0.0001) and educational status (p = 0.013), there was no significant relationship between the socio-demographic characteristics of the study participants and the levels of KOE. Other findings on the relationship between the socio-demographic characteristics and level of Knowledge of the participants were as presented in Table 3.

**Table 3:** relationship between socio-demographic variables and KOE among the entire study participants

Variable	Good knowledge Freq (%)	Poor knowledge Freq (%)	χ2	p-value
		**N=410**		
**Age**				
19-28 years	58(31.2)	128(68.8)	8.877	0.064
29-38 years	44(35.5)	80(64.5)		
39-48 years	38(50.0)	38(50.0)		
49-58 years	6(28.6)	15(71.4)		
≥59	1(33.33)	2(66.7)		
**Sex**				
Male	83(36.1)	147(63.9)	0.012	0.911
Female	64(35.6)	116(64.4)		
**Educational status^a^**				
No education	5(14.7)	29(85.3)	10.801	0.013*
Low education	38(40.0)	57(60.0)		
High education	49(43.4)	64(56.4)		
Qur’anic education	55(32.7)	113(67.3)		
**Occupational status^b^**				
Class I	0(0.0)	1(100.0)	22.061	<0.0001*
Class II	26(50.5)	26(50.0)		
Class III	47(50.9)	68(49.1)		
Class IV	28(50.9)	27(49.1)		
Class V	46(26.4)	141(75.4)		
**Marital status**				
Single	57(30.3)	131(69.7)	5.898	0.117
Married	77(40.1)	106(59.9)		
Divorced	7(30.4)	16(69.6)		
Widowed	6(37,5)	10(64.5)		

* =p<0.05, a = high education is having ≥ 12 years of western education, low education having < 12 years of western education, b = (Boroffka & Olatawura)

**Relationship between clinical variables and level of KOE:** class of epilepsy (p = 0.011), age of onset of Epilepsy (p = 0.011) and seizure frequency (p = 0.006) had significant relationship with levels of KOE. Other clinical variable assessed had no significant relationship with KOE. These findings are represented in Table 4.

**Table 4:** relationship between clinical variables and levels of KOE among the study participants

Variable	Good knowledge Freq (%)	Poor knowledge Freq (%)	χ2	P-value
		**N=410**		
**Class of Epilepsy**				
Focal	94(41.4)	129(58.6)	6.531	0.011*
Generalized	56(30.0)	131(70.0)		
**Age of onset of Epilepsy**				
Short duration (0-15) year	48(27.6)	126(72.4)	9.027	0.011*
Medium duration (16-30)	72(42.4)	98(57.6)		
Long duration >30 years	27(41.0)	39(59.0)		
**Duration of Epilepsy**				
Short duration (0-15) year	104(35.6)	183(64.4)	0.854	0.652
Medium duration (16-30)	39(36.5)	68(63.5)		
Long duration >30 years	4(33.3)	12(66.6)		
**Duration of untreated Epilepsy**				
Short duration (0-15) year	142(37.6)	236(62.4)	4.834	0.089
Medium duration (16-30)	5(17.8)	23(82.2)		
Long duration >30 years	0(0.0)	1(100.0)		
**Duration of treatment**				
Short duration (0-15) year	129(34.4)	246(65.6)	4.367	0.113
Medium duration (16-30)	16(50.0)	16(50.0)		
Long duration >30 years	2(66.7)	1(33/3)		
**Seizure frequency**				
Frequent	31(25.8)	89(74.2)	7.407	<0.006*
Less frequent	116(40.0)	174(60.0)		
**Drugs combination**				
Monotherapy	138(36.0)	245(64.0)	0.80	0.778
Polytherapy	9(33.3)	18(66.7)		

* =p<0.05 a; 0-15 years = short duration of Epilepsy, 16 - 30 years = medium duration, > 31 years is long duration, b = majority of the patients were on Carbamazepine monotherapy

**Independent predictors of KOE:** occupational statuses II (intermediate skilled) (O.R. = 2.32 p = 0.022, 95% CI = 1.13 - 4.76), III (semi-skilled) (O.R. = 1.85 p = 0.001, 95% CI = 1.10 - 3.12) IV (unskilled) (p = 0.001) and seizure frequency were independent predictors of KOE. Although educational status and class of epilepsy had a statistically significant relationship with KOE, they, however, were not independent predictors of KOE. These findings were as presented in Table 5.

**Table 5:** independent predictors of knowledge of epilepsy (KOE)

Variables	Predictor	Standard error	EXP(B) odd ratio	Confidence interval	P-values
**Knowledge of epilepsy**	Occupational status II^b^	0.366	2.32	1.13 - 4.76	0.022*
	Occupational status III^c^	0.265	1.85	1.10 - 3.12	0.001*
	Occupational status IV^d^	0.221	1.479	1.61 - 5.99	0.001*
	Seizure frequency	0.252	1.723	1.31 - 3.24	0.031*

b = intermediate skilled c = semi-skilled d = unskilled

## Discussion

**Background level of KOE among the participants:** the KOE measured at the baseline to assess participants´ level of epilepsy knowledge revealed that two-thirds of the study participants (64.15%) had lower scores than the mean scores on the EKQ and so they were adjudged to have poor KOE compared to about only one-third (35.85%) who had good KOE. This finding is in keeping with that of Mameniskiene *et al*. [1] in which they reported that PWE have poor knowledge of their disease and almost half of their study participants did not identify the cause of their illness or their type of seizures [1]. Other earlier studies have demonstrated that PWE knew little more about their disorders than the general population; regardless of their age, educational background, or number of years lived with epilepsy, while they knew more about their individual conditions than about epilepsy in general [7]. Similarly, Sunmonu *et al*. [8] in South-Western Nigeria, showed that only 10.9% of PWE had ideas on the aetiology of epilepsy [8]. Angelo [20] reported 53.3% of their study participants were found to have poor knowledge about epilepsy. Angelo *et al*. [20] also reported that PWE had a poor understanding of their disease, particularly for the underlying aetiology of their disorder. Long *et al*. [7] also discovered that PWE are not knowledgeable about their disorder regardless of age, educational background, or number of years with Epilepsy.

The reason for this poor KOE in this environment could be attributed to the low level of 'Western education' among the participants. The attribution of mental and neurological illnesses to 'jinni' (i.e. evil spirit); which is prevalent among Qur´anic and Islamic scholars and their students [21]; and the predominant traditional and cultural beliefs about the causation, presentation and treatment of Epilepsy among the indigenous people [22] may partly be responsible for the low levels of KOE. Furthermore, because of the high patient load on the clinical teams in low-resource settings such as North-Eastern Nigeria, clinicians often may not spare adequate time to psycho-educate their patients on their conditions [23]. Additionally, women have lower KOE when compared to male. The patriarchal nature of most African societies especially the setting in which this study was conducted, couple with the Islamic practice of purdah among women might have served as impediments to them learning about their condition through the process of socialization. Furthermore, the level of girl child school enrolment and education in the study environment is low compared to boy child; this could additionally contribute to this finding. On the other hand, Johnbull *et al*. [24] in Northern Nigeria, found that most of the PWE (78.6%) had adequate knowledge of AEDs. Also, they found that the level of KOE based on signs and symptoms was as high; and 57.8% and 25.9% of the PWE had excellent and good knowledge of their disease respectively [24]. this may not be surprising depending on the socio-demographic characteristics of their sample population and the fact that the report is only on the knowledge of AEDs.

**Socio-demographic predictors of KOE among the participants:** among the socio-demographic characteristics analyzed for possible association with KOE only educational status (p = 0.013) and occupational status (P <0.0001) were significantly associated with KOE. Those with higher levels of educational attainment and higher occupational class had better levels of KOE. Multivariate logistic regression however, did not reveal that educational status as an independently predict KOE (Odds Ratio, O.R., = 0.95, p = 0.652 95% C I = 0.76 - 1.18), among the participants. Occupational status on the other hand was an independent predictor of KOE. Those in class II occupation (intermediate skilled employees) were about two and half times more likely to have good KOE than unemployed (class V) (O.R. = 2.32 p = 0.022, 95% CI = 1.13 - 4.76). Also, those in class III occupations (semi-skilled employees) were about two times more likely to have good KOE than unemployed (O.R. = 1.85 p = 0.001, 95% CI = 1.10 - 3.12). Participants who were unskilled employees (O.R. = 3.11, P = 0.001, 95% C I = 1.61 - 5.99) were three times more likely to have good KOE than the unemployed. This is in harmony with the study of Angelo [20] in which they found that patients´ monthly income was a factor associated with knowledge about epilepsy. Angelo *et al*. [20] showed that higher KOE significantly correlated with a higher level of education, higher income and good seizure control for more than 2 years. They however did not find any significant correlation between levels of KOE with age, gender, and marital status.

This finding revealed that high educational status does not necessarily translate to good KOE; this means having academic qualifications does not automatically mean one is knowledgeable. Other means of getting information like the social institutions could be more instrumental in some forms of knowledge. Furthermore, being in employment may be an avenue, availed by colleagues and others, for learning about general issues including epilepsy. Excluding the only one participant who was a skilled (professional) employee and therefore belonged to class I occupation, approximately 50.0% of the participants in both class II (intermediate skilled) and IV (unskilled) of occupational status have good KOE than the remaining class of occupation. On the other hand, class V (the unemployed) had the least number of participants (3.6%) with good KOE.

**Clinical predictors of KOE among the participants:** in terms of the clinical characteristics of the participants analyzed for possible association with KOE-only class of epilepsy (p = 0.011), age of onset of Epilepsy p = 0.011 and seizure frequency (p = 0.006) were significantly associated with KOE. Those with focal epilepsy had significantly better levels of KOE than those with generalized epilepsy. Likewise, those with less frequent seizures had better KOE than those with frequent seizures. In spite of these associations with KOE however, class of epilepsy and age of onset of epilepsy as factors did not independently predict KOE (O. R. = 1.50, p = 0.076, 95% C I = 0.96 - 2.28) and (O. R. = 0.80, p = 0.47 95% C I = 0.60 - 1.08) respectively. Therefore, only seizure frequency (O. R. = 1.72, p = 0.031, 95% C I = 1.31 - 3.24) independently predicts KOE among the participants. Those with lower seizure frequencies were about two times more likely to have good KOE than those with higher seizure frequencies. This may be because patients with poor KOE may not be able to adjust and cope well with their disease. This may also affect their levels of adherence with consequent poor seizure control. This finding is affirmed by the work of Leidy *et al*. [25] in which they showed that knowledge of epilepsy correlated negatively with seizure frequency and positively correlates with self-esteem. This could further be elaborated by the fact that the more knowledgeable the PWE is about his disease condition, the more likely is he going to adhere to a treatment regimen with consequent fewer seizure frequencies. Other clinical characteristics of the participants were not associated with KOE and thus did not predict KOE. Angelo *et al*. [20] showed age at the onset of epilepsy and duration of epilepsy were found to be factors associated with knowledge about epilepsy. Angelo *et al*. [20] showed that higher KOE significantly correlated with a higher level of education, higher income and good seizure control for more than 2 years. However, they reported no significant correlation between the level of knowledge of the type of seizure, duration since diagnosis, number of AEDs, and presence of epilepsy history in the family.

**Limitations:** 1) since this is a hospital-based study conducted in one center, the findings cannot be generalized to other settings; 2) the security challenge as a result of the Boko Haram insurgency has significantly restricted access to the Maiduguri metropolis; hence, there is a low representation of other populations from other parts of the North-East geopolitical zone of Nigeria which is the catchment area of the Hospital.

## Conclusion

The level of KOE among PWE was low with more than 64.1% percent of the participants having poor KOE. Occupational status and seizure frequency were independent predictors of knowledge of epilepsy. We, therefore, recommend psycho-educational programs to be incorporated into the routine clinical care of the patients and for clinicians to pay more attention to providing information and education needs of the patients

**Recommendation:** 1) developing psycho-educational programs that will address the myths and misconceptions about the aetiology, spectra of symptoms, seizure triggers and basics of the treatment of epilepsies; 2) the active involvement of PWE and their caregivers in treatment planning and the overall management of their conditions

### What is known about this topic

- *Significant percentages of people with epilepsy do not know much about the medical aspects of their disease particularly when it comes to the aetiology, medication and outcome of the investigation, etc.;*
- *The epilepsy knowledge deficit seen among people with epilepsy does not discriminate against their age, educational background, or number of years lived with epilepsy*.

### What this study adds

- *The level of knowledge of epilepsy among its sufferers in Northeastern with peculiar low socioeconomic class and low level of education;*
- *Whether or not there is need for psycho-educational intervention for people with epilepsy*.
